# Inhibition of Quorum Sensing-Mediated Biofilm Formation and Spoilage Factors in *Pseudomonas fluorescens* by Plasma-Activated Water

**DOI:** 10.3390/foods14213773

**Published:** 2025-11-04

**Authors:** Yi-Ming Zhao, Qing-Yun Zhang, Lin Zhang, Yu-Long Bao, Yi-Ting Guo, Liu-Rong Huang, Rong-Hai He, Hai-Le Ma, Da-Wen Sun

**Affiliations:** 1 School of Food and Biological Engineering, Jiangsu University, 301 Xuefu Road, Zhenjiang 212013, China; 2 Institute of Food Physical Processing, Jiangsu University, 301 Xuefu Road, Zhenjiang 212013, China; 3 School of Biosystems and Food Engineering, University College Dublin, Belfield, D04 K1V7 Dublin, Ireland; 4 School of Food Science and Engineering, South China University of Technology, Guangzhou 510641, China

**Keywords:** plasma-activated water, quorum sensing inhibitor (QSI), AHLs degradation, biofilm inhibition, spoilage factors, *Pseudomonas fluorescens*

## Abstract

Plasma-activated water (PAW) is an emerging disinfectant; however, its potential as a quorum sensing inhibitor (QSI) for biofilm control remains underexplored, and its action mechanisms have not been elucidated. This study investigated the effects of PAW on biofilm formation and spoilage factors secretion in *Pseudomonas fluorescens* under sub-inhibitory conditions. PAW generated by treating water for 60 s (PAW-60) reduced biofilm biomass by up to 1.29 log CFU/mL after 12 h incubation. It also completely inhibited protease production (100%) and decreased siderophore production by 31.87%. N-butyryl-homoserine lactone (C_4_-HSL) was identified as the dominant signaling molecule, with its production decreasing by 34.34–84.07% following PAW treatments. Meanwhile, C_4_-HSL activity was significantly suppressed by 42.58–65.38%. An FTIR analysis revealed the formation of a new C=O group, indicating oxidative degradation of acyl homoserine lactones (AHLs). Exogenous C_4_-HSL progressively restored the biofilm biomass, spoilage factors production, and QS-related gene expression levels, with no significant difference observed compared with the control at 0.05 µg/mL (*p* < 0.05). The results suggest that the inhibitory effects of PAW are primarily due to the disruption of AHLs transduction in the QS pathway. Molecular docking showed that the long-lived reactive species in PAW could bind to AHLs’ synthetic protein (FadD1) and receptor protein (LuxR) via hydrogen bonding. PAW-60 reduced the spoilage activity of *P. fluorescens* inoculated into fish muscle juice and extended its shelf life from 8 to 10 days during storage at 4 °C. A strong positive correlation was observed between AHLs accumulation and the spoilage process. These findings demonstrate that PAW mitigates biofilm formation and food spoilage by blocking signaling transduction, which involves suppression of AHLs production, oxidative degradation of AHLs molecules, and disruption of AHLs recognition.

## 1. Introduction

Quorum sensing (QS) is a cell-to-cell communication mechanism employed by bacteria to coordinate collective behaviors in response to population density [1]. A QS system operates through the synthesis, release, and reception of signaling molecules. Upon reaching a critical threshold, signaling molecules bind to the cognate receptor and trigger the expression of target genes, thereby regulating diverse bacterial behaviors, such as bioluminescence, virulence and spoilage factors production, antibiotic resistance, biofilm formation, etc. [2]. The signaling molecules of QS are classified into three major categories: acyl homoserine lactones (AHLs) in Gram-negative bacteria, autoinducing peptides (AIPs) in Gram-positive bacteria, and autoinducer-2 (AI-2) in interspecies communication. AHLs consist of a homoserine lactone ring with a fatty acyl side chain ranging from 4 to 18 carbons (C_4_–C_18_) in length [3]. The homoserine lactone (HSL) ring is synthesized from S-adenosylmethionine (SAM), while the acyl side chain is derived either from an acyl carrier protein (ACP) or acyl-CoA precursors. The two components are conjugated by LuxI-type synthetase to form AHLs. The LuxR-type protein specifically recognizes and binds to AHLs, enabling target gene expression in a cell-density-dependent manner. Quorum sensing inhibitors (QSIs) disrupt bacterial communication by targeting distinct stages of the QS system and are broadly categorized into three classes: (1) inhibition of signal molecular synthesis by interfering with the LuxI-type protein, as observed with certain natural extracts [4]; (2) signal degradation via structural modification, such as quorum quenching (QQ) enzymes [5]; and (3) blockage of QS signal reception through disruption of LuxR-type protein or ligand recognition [6]. Among the QQ enzymes, three major types are distinguished based on the catalytic sites on AHLs: AHL lactonases can catalyze and open the homoserine lactone ring; AHL acylases hydrolyze the amide bound between the lactone ring and the acyl chain; and AHL oxidoreductase modifies the acyl chain thorough oxidation or reduction. Biofilms are structured microbial communities encased with a self-produced extracellular polymeric matrix. Compared to planktonic bacteria, biofilms significantly enhance resistance to environmental stresses, including chemical disinfectants, antibiotics, thermal fluctuations, etc., rendering them remarkably difficult to eradicate [7]. Excessive use of antibiotics or chemical sanitizers has resulted in the emergence of multidrug-resistant bacteria, which is a major public health concern [8]. Recent studies have demonstrated that quorum sensing inhibitors (QSIs) can suppress biofilm development through disrupting the QS system without directly inactivating or killing bacteria [9,10,11]. Therefore, QSIs can reduce the risk of inducing microbial resistance. This attribute provides an outstanding advantage over conventional antibiotics and chemical sanitizers, which exert selective pressure through bactericidal action [12].

*Pseudomonas fluorescens* has been identified as a specific spoilage organism (SSO), especially in protein-rich foods, such as aquatic, meat, and dairy products [10]. *P. fluorescens* exhibits potent spoilage activity through extracellular secretion of protease, lipase, and siderophore. These spoilage factors decompose food into amines, ammonia, aldehydes, sulfides, free fatty acids, etc., causing rapid quality degradation and offensive off-flavors [13]. Chen et al. [14] demonstrated the important role of siderophores in biofilm formation and the spoilage potential of *P. fluorescens* via constructing deletion mutation of siderophore biosynthesis genes (*pvdA* and *pvdE*). Consequently, spoilage factor production is also a particular concern [15]. In addition, as a psychrophilic bacterium, *P. fluorescens* maintains its metabolic activity and cellular replication even at refrigeration temperatures, posing significant challenges for food quality and safety [16]. Previous studies have shown that the spoilage ability of *P. fluorescens* is closely related to the QS system [17,18,19]. AHLs are the common QS signaling molecules in *P. fluorescens*. Therefore, targeting AHLs in *P. fluorescens* could be a promising strategy for food spoilage control.

Plasma, a partially or wholly ionized gas, composed of reactive species, charged particles, neutral atoms, etc. [20], is commonly categorized into non-thermal (cold) and thermal types. The former is particularly suitable for laboratory applications due to its low-temperature operation. Plasma-activated water (PAW) is generated by exposing water to cold plasma discharge. During this process, the plasma–water interactions initiate a series of chemical reactions that lead to the generation of reactive oxygen species (ROS) and reactive nitrogen species (RNS), which endow PAW with distinctive physicochemical properties [21,22,23,24]. PAW has shown significant antibacterial effects and is considered a potential green disinfectant. The ability of plasma and PAW to inhibit biofilm formation has been reported [25,26,27], and while most studies have focused on its bactericidal effects under lethal conditions, its capacity to act as a QSI remains largely unexplored. Cai et al. [28] found that AHLs production and biofilm formation of *P. aeruginosa* were reduced after PAW treatment. Li et al. [29] showed that PAW inhibited biofilm formation and downregulated the QS-related virulence gene expression of *Enterococcus faecalis*. However, the QS pathway involves multiple stages, including the production, release, and recognition of signaling molecules. To the best of our knowledge, the action mechanisms of PAW as a QSI against biofilm formation and spoilage factor production have not been reported, and the relationship between the QS signaling molecules and the spoilage process of food has not been illustrated.

In this study, the effects of PAW on the biofilm formation and spoilage factors secretion of *P. fluorescens* under sub-inhibitory conditions were investigated. To reveal the action mechanisms of PAW as a QSI, the dominant QS signaling molecule in *P. fluorescens* was identified using high-performance liquid chromatography (HPLC), and the effects of the selected PAW treatments on AHLs production, activity, and structure were examined. The impacts of exogenous AHLs supplementation on biofilm formation, spoilage factors, and QS-related gene expression levels were measured. In addition, the molecular docking interactions between the long-lived reactive species in PAW and the critical QS pathway proteins were investigated. Finally, PAW was applied for food preservation via inoculating *P. fluorescens* into fish muscle juice and storing it at 4 °C for 10 days. During storage, the total viable counts (TVCs), pH, thiobarbituric acid reactive substances (TBARS), total volatile basic nitrogen (TVB-N), and AHLs production were measured.

## 2. Materials and Methods

### 2.1. Strains and Culture Preparation

The *P. fluorescens* employed in this investigation was originally isolated from a large yellow croaker, which was marked as PF14. The biosensor strains of *Agrobacterium tumefaciens* KYC55 (KYC55) and *Chromobacterium violaceum* 026 (CV026) were kindly provided by Prof. Mingsheng Dong (Nanjing Agricultural University, Nanjing, China) and Prof. Junli Zhu (Zhejiang Gongshang University, Hangzhou, China), respectively. Neither KYC55 nor CV026 produces AHLs, but they can sense exogenous AHLs to produce β-galactosidase and violacein, respectively. PF14 was overnight-cultured in tryptic soy broth (TSB, HangZhouBaiSi Biochemical Technology Co., Ltd., Hangzhou, China) at 28 °C. Following incubation, the bacterial cells were harvested via centrifugation at 12,000× *g* for 5 min using a high-speed centrifuge (TGL 18M, Yancheng Kaite Experimental Instrument Co., Ltd., Yangcheng, China), the supernatant was removed, and the pellet was resuspended in sterile deionized water to the initial culture volume.

### 2.2. PAW Generation Under Sub-Inhibitory Conditions

For the PAW generation, an atmospheric plasma jet (PG-1000ZD, Nanjing Suman Plasma Technology Co., Ltd., Nanjing, China) was employed. The plasma system was operated at 300 W and 20 kHz with a 30 L/min compressed air flow. The plasma nozzle was positioned 10 cm above the water surface during generation. The PAW was obtained by exposing 200 mL of sterile deionized water to the plasma jet for varying durations (30, 40, 50, and 60 s), which were designated as PAW-30 through PAW-60. Our previous study showed that no significant inhibitory effect on bacterial counts or growth curves was observed after selected PAW treatments [30], indicating that the PAW treatments were under sub-inhibitory conditions of PF14. For the PAW treatment, 1 mL of bacterial suspension was transferred to 9 mL of freshly prepared PAW, and the contact time of the mixture was 2 min. The bacterial suspension treated with sterile deionized water was used as the control.

### 2.3. Enumeration of Bacterial Cells in Biofilm

Biofilm biomass of PF14 was quantified based on Li et al. [31]. A sterile glass slice (20 mm × 20 mm) was placed in a 6-well microplate, then 0.5 mL of PAW-treated bacterial suspension was 1:10 (*v*/*v*) inoculated to TSB in each well before incubation at 28 °C for 12, 24, 48, and 72 h. The glass slice was rinsed thrice using 0.01% PBS (Hunan Bikemam Biotechnology Co., Ltd., Changsha, China) after incubation. Each slice was transferred to a sterilized beaker containing 10 mL of 0.01% PBS and subjected to ultrasonication (Ymnl-1000Y, Nanjing Immanuel Instrument Equipment Co., Ltd., Nanjing, China) at 40 kHz/L (40 W) for 10 min. The detached bacteria were decimally diluted in 0.1% (*w*/*v*) peptone water (Guangdong Huankai Microbial SCI&TECH. Co., Ltd., Guangzhou, China) and plated on tryptic soy agar (TSA, HangZhouBaiSi Biochemical Technology Co., Ltd., Hangzhou, China). The colonies between 30 and 300 were counted after incubation at 28 °C for 48 h.

### 2.4. Spoilage Factors Assay

Protease and siderophore are the main spoilage factors of *P. fluorescens*, which were determined by the plate perforation method [32]. PF14 was incubated at 28 °C for 12, 24, 48, and 72 h, respectively, after the PAW treatment described in Section 2.2. Cell-free supernatants were obtained by centrifugation (12,000× *g*, 5 min) and sterile filtration (0.22 μm filter). The production of protease and siderophore was measured using plates containing 1.5% (*v*/*v*) skimmed milk and Chrome Azurol Sulphonate (CAS) plates (Qingdao Hi-Tech Industrial Park Hope bio Technology Co., Ltd., Qingdao, China), respectively. The wells in the plates were punched using a sterilized Oxford cup, and 200 µL of the supernatant was pipetted into the wells. After incubation at 28 °C for 24 h, the diameters of the transparent hydrolysis circle and the orange circle were measured as the protease and siderophore production, respectively.

### 2.5. AHLs Production Assay

The type of AHLs in *P. fluorescens* and the effect of PAW on the production of AHLs were determined using HPLC. As described in Section 2.2, 3 mL of the PAW-treated bacterial suspension was transferred into 27 mL of TSB and incubated at 28 °C for 24 h. The bacterial suspension treated with sterile deionized water was used as the control. The AHLs were extracted according to the method of Li et al. [19] with slight modifications. The culture was centrifuged (12,000× *g*, 5 min) after incubation, and the supernatants were extracted three times with an equal volume of ethyl acetate with 0.1% (*v*/*v*) acetic acid. The extracts were evaporated to dryness in a rotary evaporator at 30 °C, and the residue was dissolved in 1 mL of methanol and passed through a 0.22 μm sterile filter. Standard solutions of C_4_-HSL, C_6_-HSL, C_8_-HSL, C_10_-HSL, C_12_-HSL, and C_14_-HSL (Sigma Aldrich, St. Louis, MO, USA) were prepared to determine the type and production of AHLs. The extracts and standard solutions were analyzed using an HPLC system (Thermo Fisher Scientific Co., Ltd., Waltham, MA, USA), which was equipped with Aminex HPLC Columns (Bio-Rad Laboratories, Inc., Hercules, CA, USA) and a UV detector at 210 nm. The mobile phase consisted of water (solvent A) and methanol (solvent B), both of which contained 0.1% formic acid. The gradient elution procedure was performed as follows: 0–20 min, 70% B; 20–40 min, 90% B; 40–60 min, 70% B [33].

### 2.6. AHLs Activity Assay

Exogenous AHLs can induce KYC55 to express β-galactoside, which can hydrolyze o-nitro-β-d-galactoside (ONPG) to yellow ortho-nitrophenol [34]; thus, AHLs activity can be reflected by measuring the β-galactoside activity. The AHLs activity was determined according to the method of Xu et al. [35] with slight modifications. KYC55 was cultured overnight (28 °C, 16 h) in TSB supplemented with 1 µg/mL of tetracycline, 100 µg/mL of gentamicin, and 100 µg/mL of spectinomycin. The AHLs crude was prepared as described in Section 2.5. A total of 200 μL of the crude was pipetted into 2 mL of TSB containing 1% (*v*/*v*) overnight-cultured KYC55 and incubated at 28 °C for 16 h, followed by measurement of OD_600_ using a spectrophotometer (Thermo Fisher Scientific Co., Ltd., Waltham, USA). A total of 0.9 mL of Z-buffer, 45 μL of chloroform, and 15 μL of 0.1% SDS were added to a 5 mL sterile centrifuge tube containing 100 μL of KYC55 bacterial suspension and mixed thoroughly. Then, 200 μL of 4 mg/mL ONPG was transferred to the centrifuge tube, and the mixture was incubated at 28 °C after mixing. A total of 600 μL of 1 M Na_2_CO_3_ was pipetted immediately into the mixture to terminate the reaction when the solution turned yellow, and the time (T) the solution took to change color was recorded. Finally, the absorbance of the supernatant at 420 nm was measured after centrifugation (12,000× *g*, 5 min). The β-galactosidase activity was calculated using the following formula:

β-galactosidase activity = (1000 × OD_420_)/(OD_600_ × T × V) (1)

where OD_420_ or OD_600_ means the absorbance of the solution at 420 or 600 nm. T means the time (min) the solution took to change color to yellow. V means the volume (mL) of the crude AHLs extracts, which was 0.2 in our study.

### 2.7. AHLs Structure Assay

FTIR was used to investigate the impact of PAW treatment on the AHLs structure, which was determined by referring to the method of Flynn et al. [36] with slight modifications. A total of 0.1 mL of 1 mg/mL AHLs was pipetted into 0.9 mL of PAW, and the spectra were measured at 4000–400 cm^−1^ after 2 min exposure using an FTIR (Nicolet iS50, Thermo Fisher Scientific Co., Ltd., Waltham, USA) with a resolution of 32 cm^−1^ and an average of 32 scans. A total of 100 μg/mL of the standard AHLs solution was used as the control.

### 2.8. Determination of Biofilm Formation with Exogenous C_4_-HSL

The effect of exogenous C_4_-HSL on the biofilm formation of PF14 after the PAW-60 treatment was determined by the crystal violet staining method [25]. In brief, a sterilized glass slice (20 mm × 20 mm) served as the biofilm carrier and was placed in a 6-well plate. Each well was immersed with 4.5 mL of TSB containing different concentrations of C_4_-HSL (0, 0.01, 0.05, 0.1, and 0.2 µg/mL); then, 0.5 mL of bacterial suspension was transferred to the wells after the PAW-60 treatment and incubated at 28 °C for 24 h. The bacterial suspension treated with sterile deionized water served as the control. After incubation, the biofilm on the glass slide was rinsed three times using 0.01% PBS to remove planktonic bacteria, and stained with 0.5% crystal violet for 10 min; then, the unbound crystal violet was washed off with 0.01% PBS. Finally, 95% ethanol was transferred to dissolve the residual crystal violet, and the absorbance at 590 nm was measured.

### 2.9. Determination of Spoilage Factors with Exogenous C_4_-HSL

The protease and siderophore production were determined by the plate perforation method. As described in Section 2.8, PF14 was incubated in TSB containing different concentrations of C_4_-HSL (0, 0.01, 0.05, 0.1, and 0.2 µg/mL) for 24 h after PAW-60 treatment. The production of protease and siderophore was determined as described in Section 2.4.

### 2.10. Determination of Gene Expression with Exogenous C_4_-HSL

Four QS-regulated genes (biofilm formation, *SgaS*; spoilage factors: *TolC*, *pvdA*; AHLs receptor: *LuxR*) were selected based on the transcriptomic annotation. The selected gene expression was determined by RT-qPCR. PF14 was incubated at 28 °C for 24 h after PAW-60 treatment according to Section 2.8. The supernatant was removed after centrifugation at 12,000× *g* for 5 min at 4 °C, and the pellets were collected. The bacterial RNA was extracted according to the instructions of the RNA Extraction Kit (Sangon Biotech Co., Ltd., Shanghai, China). The cDNA was synthesized using a HisyGo RT Red SuperMix for qPCR (+gDNA Wiper) kit (Vazyme, Nanjing, China). An RT-qPCR analysis was performed according to the SYBR Green I kit (Vazyme, Nanjing, China). 16S rRNA of PF14 was used as the internal reference gene, and the primers used in this study are listed in Table 1.

### 2.11. Molecular Docking

The action mechanisms between the long-lived reactive species (H_2_O_2_, NO_2_^−^, and NO_3_^−^) in the PAW and key biomolecules (acyl-CoA synthetase of AHLs, FadD1; AHLs receptor, LuxR) were studied by molecular docking. The biomolecules (FadD1, LuxR) were selected based on our previous transcriptomic results, which demonstrated that the genes were downregulated after PAW-60 treatment [30]. Swissmodl platform (http://swissmodel.expasy.org/, accessed on 24 October 2025) was used to predict the molecular structure of proteins, and the water molecules and metal ions of proteins were removed using PyMOL 2.5.2. The molecular docking was performed using AutoDock Vina program (http://vina.scripps.edu/, accessed on 24 October 2025), and the results were visualized using Py MOL 2.5.2.

### 2.12. In Vivo Spoilage Potential of P. fluorescens in Fish Muscle Juice Assay

To evaluate the effect of PAW on the spoilage potential of *P. fluorescens*, PF14 was inoculated into sterile fish muscle juice after a PAW-60 treatment and stored at 4 °C for 10 days, during which the TVC, pH, TBARS, TVB-N, and AHLs production in the fish muscle juice were analyzed every 2 days until day 10.

#### 2.12.1. Fish Muscle Juice Contamination

Large yellow croakers were purchased from Metro supermarket (Zhenjiang, China) and transported to the laboratory on ice within 1 h. Sterile fish muscle juice was prepared according to the method of Dalgaard [37]. Briefly, the fish were cleaned, minced, and boiled in an appropriate amount of sterilized water for 5 min. The fish muscle juice was obtained by filtration, centrifugation, and sterilization at 121 °C for 15 min. Then, trimethylamine oxide, L-cysteine, and L-methionine were added to the fish muscle juice to reach a final concentration of 1.6, 40, and 40 mg/L, respectively, to compensate for the nutrient loss caused by dilution and sterilization. After the PAW-60 treatment, as described in Section 2.2, the mixture was immediately tenfold diluted to achieve a concentration of 3–4 log CFU/mL. Then, 5 mL of the diluted solution was transferred into 45 mL of sterile fish muscle juice to reach an inoculated level of 2–3 log CFU/mL and stored at 4 °C for 10 days. The bacteria treated with sterile deionized water were used as the control. During storage, 10% (*v*/*v*) fresh PAW-60 or sterile deionized water was added to the fish muscle juice at 12 h intervals.

#### 2.12.2. TVC and pH Analysis

The TVC of the fish muscle juice was determined by the standard plate counting method according to Liu et al. [38]. In brief, 1 mL of the fish juice was transferred into 9 mL of sterile water and tenfold diluted using 0.1% (*w*/*v*) peptone water. A total of 100 μL of appropriate dilution was plated on the TSA, and the colonies were counted after incubation at 28 °C for 48 h. The pH of the fish muscle juice was determined using a pH meter (LE438, Mettler-Toledo International Inc., Zurich, Switzerland).

#### 2.12.3. TBARS and TVB-N Analysis

TBARS of the fish muscle juice was detected according to Gan et al. [39] with slight modifications. A total of 5 mL of the fish muscle juice was mixed thoroughly with 50 mL of trichloroacetic acid and filtered using Whatman filter paper. After that, 5 mL of the filtrate was mixed with 5 mL of 0.02 M thiobarbituric acid (TBA). The mixture was vortexed thoroughly before being subjected to a water bath (90 °C, 30 min). The absorbance of the solution was measured at 590 nm after cooling down to room temperature. A standard curve of malondialdehyde (MDA) was plotted to transform the concentration into absorbance values. The TBARS was expressed as mg MDA/100 mL. The TVB-N was quantified according to the Chinese National Standard GB 5009.228–2016. In brief, 25 mL of the fish muscle juice was homogenized with 100 mL of deionized water and filtered after 30 min. The filtrate was acidified with 10 mL of perchloric acid, and the basic nitrogen compounds were steam-distilled before titration with 0.01 M HCl. TVB-N of the fish muscle juice was expressed as mg N per 100 mL.

#### 2.12.4. AHLs Production Analysis

The biosensor strain of CV026 does not produce AHLs, but it can sense exogenous AHLs to produce violacein, and violacein can dissolve in dimethylsulfoxide (DMSO), and thus the absorbance of violacein can semi-quantitatively determine AHLs production. The AHLs secreted by PF14 into the fish muscle juice were detected using CV026 according to the method of Li et al. [40] with some modifications. A total of 20 mL of the fish muscle juice was centrifuged (12,000× *g*, 5 min), and the AHLs in the supernatants were extracted as described in Section 2.5. The crude extract was dissolved in methanol and stored at −20 °C for later use. CV026 was inoculated into TSB supplemented with 20 µg/mL kanamycin and incubated at 28 °C for 16 h. A total of 200 μL of the AHLs extracts was transferred into 5 mL of fresh TSB inoculated with 1% (*v*/*v*) overnight-cultured CV026. After incubation at 28 °C for 24 h, 1 mL of the solution was centrifuged (12,000× *g*, 5 min), and the pellets were resuspended in 1 mL of DMSO. The suspension was vortexed thoroughly to allow for the full dissolution of violacein before centrifugation (12,000× *g*, 5 min), and the absorbance of the supernatant was detected at 590 nm using a spectrophotometer (Thermo Fisher Scientific Co., Ltd., Waltham, MA, USA).

### 2.13. Statistical Analysis

In this study, each group of experiments was performed in triplicate, and the values were expressed as the mean ± standard deviation. SPSS 16.0 software was used for the statistical analysis. An analysis of variance (ANOVA) was used to evaluate the differences between groups, and a level of *p* < 0.05 was considered statistically significant. In addition, Pearson correlation heatmaps between the PAW generation time and biofilm formation, spoilage factor production, AHLs production, and spoilage parameters of fish muscle juice were visualized using SPSS.

## 3. Results and Discussion

### 3.1. The Effect of PAW on Biofilm Biomass

**Figure 1:**
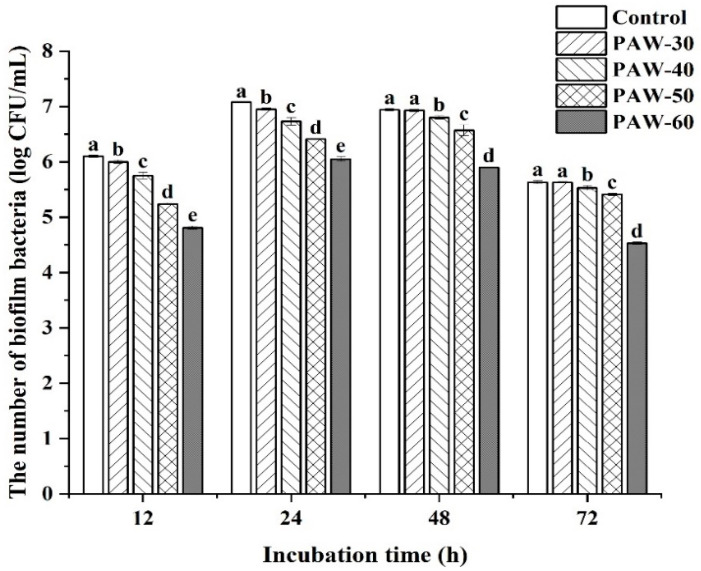
Enumeration of biofilm biomass in PF14 after PAW treatments over 72 h incubation. The results are presented as mean ± SD (*n* = 3). Different letters indicate significant differences (*p* ˂ 0.05).

Biofilm formation of PF14 during the 72 h incubation was quantitatively analyzed by calculating the viable counts in the biofilm. As shown in Figure 1, all the selected PAW treatments decreased biofilm formation compared to the control, with a significant difference observed after incubation for 12 and 24 h (*p* < 0.05), and the biofilm formation was negatively correlated to the PAW generation time (shown in Figure 3). The biofilm at 12 h incubation was significantly decreased by 0.10, 0.35, 0.86, and 1.29 log CFU/mL, respectively, after PAW-30, PAW-40, PAW-50, and PAW-60 treatments (*p* < 0.05). Moreover, Figure 1 shows that biofilm formation reached a maximal level at 24 h before a subsequent decline until 72 h. Thus, the peak biofilm was obtained at 24 h, with values of 7.08, 6.95, 6.73, 6.41, and 6.05 Log CFU/mL for the control, PAW-30, PAW-40, PAW-50, and PAW-60, respectively. The trend in the biofilm biomass can be attributed to the maturation phase within 24 h, followed by the dispersal phase from 24 to 72 h. The strengthened inhibitory effect resulted from the accumulated reactive species in the PAW when the PAW generation time was extended [41], which enhanced disruption of the QS pathway and led to suppression of biofilm formation. A previous study also showed that PAW treatments mitigated biofilm biomass of *Enterococcus faecalis* in an ultra-low-dose ROS group during a 72 h incubation, and no inhibitory effect against planktonic bacteria was observed at 0 h [30]. Li, Wang [20] reported that a sub-minimum inhibitory concentration (MIC) of cinnamaldehyde (0.025 to 0.1 μL/mL) reduced biofilm formation in *P. fluorescens* by 35.74–54.48%. Zhang et al. [42] showed that a sub-MIC of hexanal, an essential oil component, significantly suppressed biofilm formation in *P. fluorescens* and *Erwinia carotovora*, with a dose-dependent inhibition pattern observed across concentrations during 1–3 days incubation.

### 3.2. The Effect of PAW on Spoilage Factors

**Figure 2:**
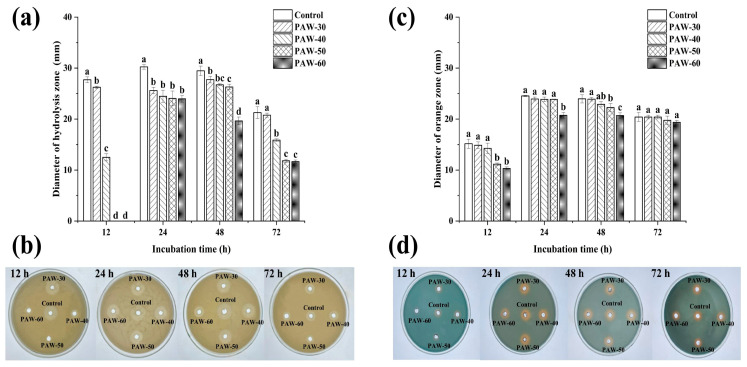
The effect of PAW on spoilage factors of (**a**,**b**) protease and (**c**,**d**) siderophore in PF14 during 72 h incubation. The results are presented as mean ± SD (*n* = 3). Different letters indicate significant differences (*p* ˂ 0.05).

**Figure 3:**
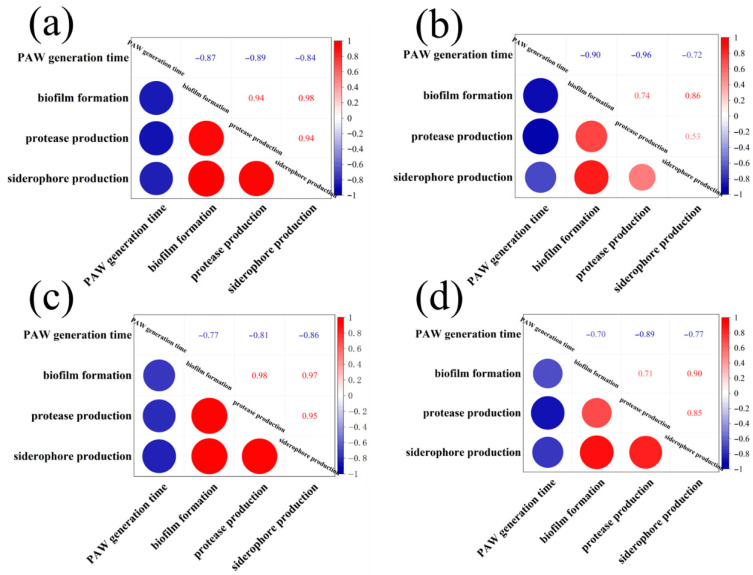
Pearson correlation between PAW generation time and biofilm formation, protease production, and siderophore production at different incubation times: (**a**) 12 h, (**b**) 24 h, (**c**) 48 h, (**d**) 72 h.

Similar to biofilm formation, the secretion of spoilage factors is also a bacterial behavior regulated by the QS system. Proteases can degrade proteins into sulfur-containing compounds and volatile nitrogen metabolites, leading to food spoilage. Siderophore is a small molecule with specific chelation of iron ion (III), which enables bacteria to acquire iron under nutrient-limited conditions and improve their survival competitiveness [17]. Therefore, spoilage factors represent another major determinant of spoilage potential. As presented in Figure 2a,b, the production of protease and siderophore decreased after the PAW treatments, and the suppression rates were enhanced with prolonged PAW generation times. Figure 2a shows that the diameter of the hydrolysis circle at 12 h incubation was significantly decreased from 27.70 to 26.25 and 12.50 mm after the PAW-30 and PAW-40 treatments, respectively (*p* < 0.05), and no hydrolysis circle was observed after the PAW-50 and PAW-60 treatments, indicating a complete inhibition of protease production. However, there was no significant difference in siderophore production at 12 h incubation after the PAW-30 and PAW-40 treatments, and a significant reduction of 26.37% and 31.87% was obtained after the PAW-50 and PAW-60 treatments, respectively (*p* < 0.05). Similar to biofilm formation, both protease and siderophore production peaked at 24 h and then decreased until 72 h, which is consistent with previous studies [14,43]. In addition, Figure 3 shows that biofilm formation, protease production, and siderophore production were positively correlated with each other throughout the 72 h incubation. As the spoilage factor production is regulated by the QS system via a series of cascading reactions [18], the decrease in the spoilage factor production can be attributed to the disruption of the QS pathway after the PAW treatment. Though to the best of our knowledge, a study of PAW on spoilage factor production has not been conducted, the effects of many other antibacterial substances have been reported [19,44,45]. Li, et al., [46] found that the protease production of *P. fluorescens* decreased by 58.50% under a sub-MIC concentration of 0.1 µg/mL cinnamaldehyde. Wang et al., [17] showed that the siderophore production of *P. fluorescens* decreased after a Cytidine-5′-monophosphate (5′-CMP) and 5′-adenylic acid treatment.

### 3.3. The Effect of PAW on AHLs Production of PF14

**Figure 4:**
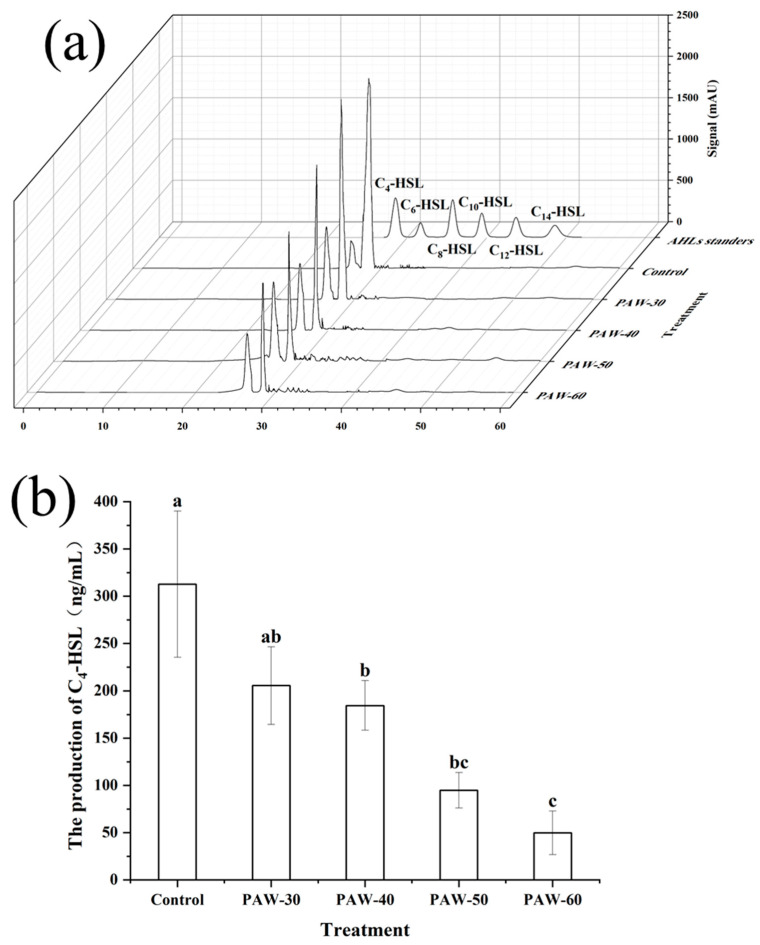
(**a**) HPLC chromatogram of standard AHLs and AHLs types in PF14. (**b**) The effect of PAW on AHLs production of PF14. The results are presented as mean ± SD (*n* = 3). Different letters indicate significant differences (*p* ˂ 0.05).

To reveal the action mechanisms of PAW as a QSI against biofilm formation and spoilage factors production in *P. fluorescens*, the types of AHLs produced by PF14 were first identified by comparing them with AHLs standards. As shown in Figure 4a, C_4_-HSL was identified as the most dominant AHL after incubation for 24 h. The type of AHLs is consistent with the previous study of Tang et al. [18], who also detected C_4_-HSL as the main AHLs in *P. fluorescens* after 24 h incubation. Furthermore, the production of C_4_-HSL remarkably decreased, from 312.80 to 205.39, 184.47, 94.78, and 49.82 µg/mL, respectively, after the PAW-30, PAW-40, PAW-50, and PAW-60 treatments, with a reduction of 34.34%, 41.03%, 69.70%, and 84.07%, respectively (Figure 4b). The decrease in AHLs production indicates that QS signaling production was disrupted after PAW treatment. Since biofilm formation and spoilage factor production are QS-regulated phenotypes, the disruption of the QS pathway caused a reduction in the biofilm biomass and spoilage factor production, which was demonstrated in Section 3.1 and 3.2, respectively. The study of Cai et al. [28] demonstrated a 42.00% and 100% (undetectable level) reduction in C_4_-HSL production in *P. aeruginosa* after PAW and plasma-activated lactic acid (PALA) treatments, respectively. Flynn et al. [36] showed that no violacein production in the bio-reporter CV026 was observed after AHLs were exposed to plasma for 60 s, indicating complete decomposition. Yin et al. [11] also reported a significant decrease in C_4_-HSL in *P. aeruginosa* after exposure to sub-MICs of soy isoflavones, and a dose-dependent manner was observed. Fidaleo et al. [47] revealed that triclosan inhibited overall AHLs production by preventing the activity of acyl carrier protein reductase FabL protein.

### 3.4. The Effect of PAW on AHLs Activity of PF14

**Figure 5:**
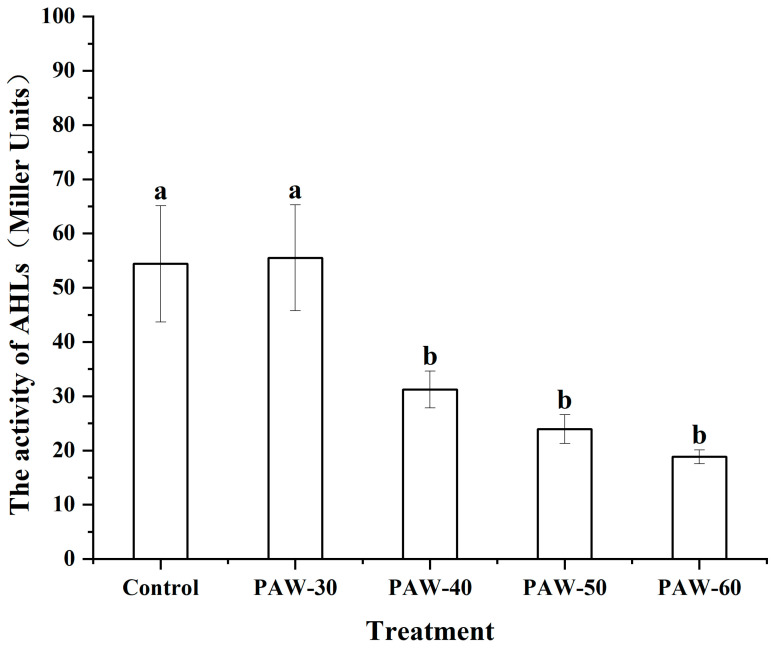
The effect of PAW on AHLs activity of PF14. The results are presented as mean ± SD (*n* = 3). Different letters indicate significant differences (*p* ˂ 0.05).

As illustrated in Figure 5, the PAW treatments attenuated AHLs activity, and the PAW-60 showed the maximal repression. There was no significant reduction in the activity of AHLs after the PAW-30 treatment compared to the control, while PAW-40, PAW-50, and PAW-60 significantly reduced the activity of AHLs by 42.58%, 55.99%, and 65.38%, respectively (*p* < 0.05). The weakened activity of AHLs resulted from oxidative degradation by the reactive species generated in the PAW. Though studies of PAW on AHLs activity are quite rare, the effects of other antibacterial substances on AHLs activity have been reported. Zhang et al. [48] showed that the activity of AHLs in *P. fluorescens* and *Erwinia carotovora* decreased during monitoring over 24 h under different sub-MICs of hexanal. Zhao et al. [49] found that the AHLs activity of *Aeromonas vickerii* was reduced by 39.60% when the concentration of garlic extract was 1.20 mg/mL. Frey et al. [50] also reported decreased AHLs activity after a hydroxyl radical treatment. Shen et al. [51] discovered that PF-1240 (a new QQ enzyme) reduced AHLs activity via quenching AHLs with different carbon chain lengths.

### 3.5. The Effect of PAW on C_4_-HSL Structure

**Figure 6:**
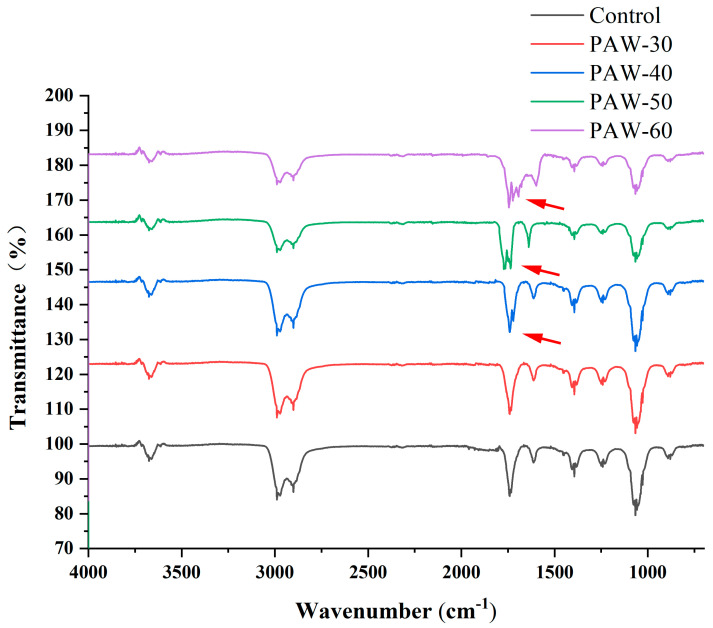
The FTIR spectra of C_4_-HSL after PAW treatments. The red arrow indicate the generation of C=O group.

According to Section 3.3, the dominant signaling molecule of PF14 is C_4_-HSL, and thus C_4_-HSL was selected as the subject of this study. As shown in Figure 6, PAW-30 did not change the structure of C_4_-HSL compared with the standard. A vibration at approximately 1710 cm^−^ was observed after the PAW-40, PAW-50, and PAW-60 treatments, indicating the generation of a new C=O group in the C_4_-HSL [36]. The vibration of the new functional group gradually strengthened with a prolonged PAW generation time, suggesting that C_4_-HSL oxidation was enhanced. The acyl side chain of C_4_-HSL underwent oxidative modification via the ROS in the PAW, leading to structural alterations and compromised signaling potency. PAW-30 did not cause structural changes in C_4_-HSL, which explains the maintenance of AHLs activity in Section 3.4. PAW-60 exhibited the most pronounced oxidative degradation to C_4_-HSL, thereby causing the greatest reduction in C_4_-HSL activity (Section 3.4). Flynn et al. [36] reported that an OH functional group and a new C=O group were generated after four types of AHLs were exposed to plasma for 60 s using UHPLC-MS, and complete decomposition after 240 s exposure. They also found that shorter-chain AHLs were more resistant to plasma degradation than longer-chain AHLs. Chowdhary et al. [52] reported that CYP102A1, a well-known cytochrome from *Bacillus megaterium*, efficiently oxidized AHLs, and the oxidation mainly happened at the ω-1, ω-2, and ω-3 carbons of the acyl side chain.

### 3.6. The Effect of Exogenous C_4_-HSL on the Biofilm Formation of PF14

**Figure 7:**
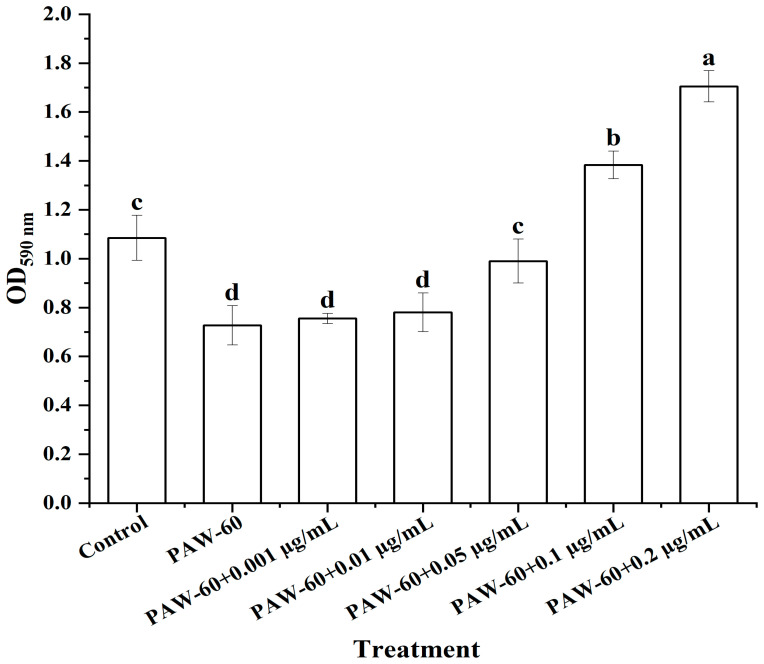
The effect of exogenous C_4_-HSL on the biofilm formation of PF14 after PAW-60 treatment. The results are presented as mean ± SD (*n* = 3). Different letters indicate significant differences (*p* ˂ 0.05).

As shown in Figure 7, PAW-60 significantly reduced the biofilm biomass of PF14 by 33.03% after 24 h of incubation compared with the control (*p* < 0.05). The biofilm biomass of PF14 was increased with the supplementation of exogenous C_4_-HSL in a dose-dependent manner, demonstrating the vital role of the QS signaling molecule in biofilm formation. There was no significant difference compared with the control when the exogenous C_4_-HSL was 0.05 µg/mL, and the biofilm biomass was significantly increased by 26.60% and 56.88%, respectively, when the exogenous supplementation was 0.1 and 0.2 µg/mL (*p* < 0.05). Li et al. [29] reported that the biofilm formation of *P. fluorescens* was significantly increased by 127.49% when the exogenous C_4_-HSL was 2 µg/mL (*p* < 0.05). The findings further indicated that PAW treatment suppressed biofilm formation by disrupting QS signaling transduction, and the supplementation of exogenous C_4_-HSL could restore biofilm formation.

### 3.7. The Effect of Exogenous C_4_-HSL on the Spoilage Factors of PF14

**Figure 8:**
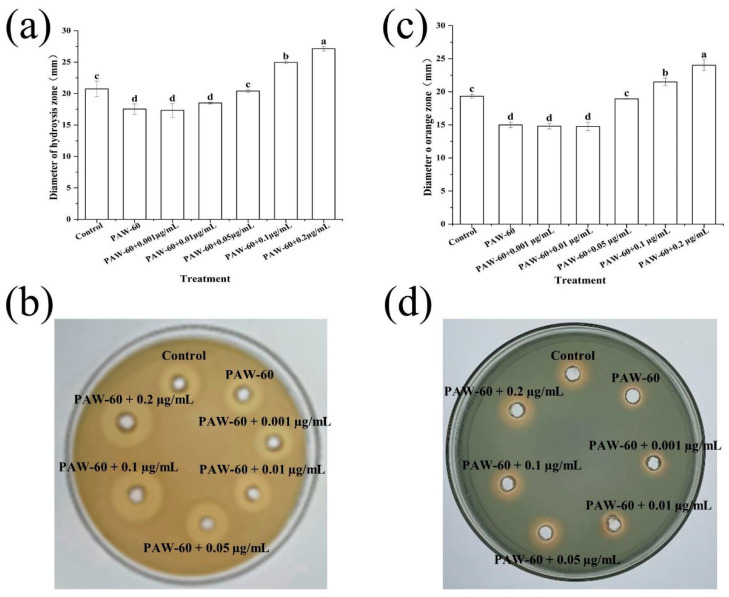
The effect of exogenous C_4_-HSL on (**a**,**b**) protease and (**c**,**d**) siderophile production of PF14 after PAW-60 treatment. The results are presented as mean ± SD (*n* = 3). Different letters indicate significant differences (*p* ˂ 0.05).

As shown in Figure 8, both protease (a,b) and siderophore (c,d) production were gradually restored with the supplementation of exogenous C_4_-HSL. Similar to biofilm formation, there was no significant difference in protease and siderophore production compared with the control when the exogenous C_4_-HSL was 0.05 µg/mL. The protease and siderophore production increased significantly, by 30.86% and 24.31%, respectively, with the maximum supplementation of 0.2 µg/mL (*p* < 0.05). The results indicate that AHLs modulate spoilage factor production in *P. fluorescens*, and PAW acts as a disruptor of AHLs transduction. Li et al. [29] showed that 2 µg/mL of exogenous C_4_-HSL stimulated the protease production of *P. fluorescens*. Zhang et al. [53] reported that 100 ng/mL of exogenous C_8_-HSL resulted in a 59.68% recovery of protease activity in *P. fluorescens* after being treated with sub-MIC hexanal.

### 3.8. The Effect of Exogenous C_4_-HSL on the Gene Expression of PF14

**Figure 9:**
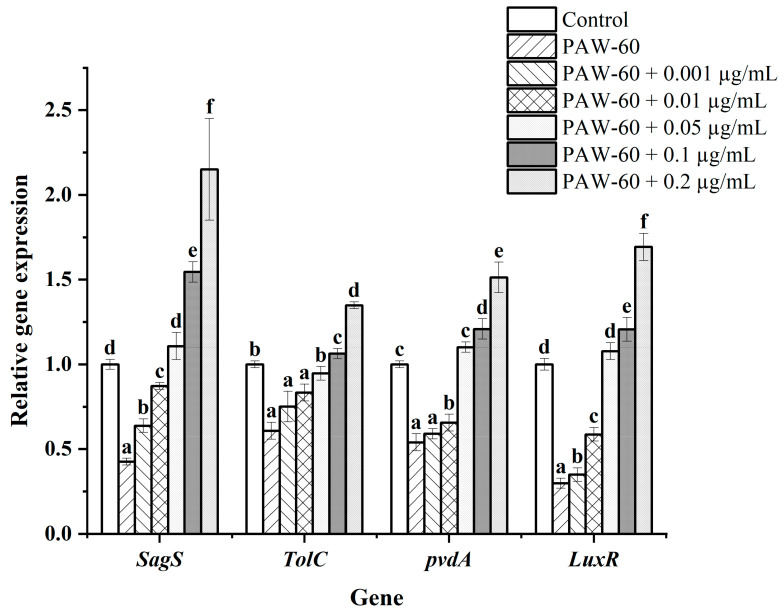
The effect of exogenous C_4_-HSL on gene expression levels of PF14 after PAW-60 treatment. The results are presented as mean ± SD (*n* = 3). Different letters indicate significant differences (*p* ˂ 0.05).

Gene transcription was determined using RT-qPCR. As shown in Figure 9, the genes involved in biofilm formation (*SgaS*), spoilage factors (*TolC*, *pvdA*), and AHLs receptor (*LuxR*) were all downregulated after the PAW-60 treatment, and supplementation with exogenous C_4_-HSL stimulated gene expression in a dose-dependent manner. There was no significant difference in gene expression of biofilm formation and spoilage factor production compared with the control when the exogenous C_4_-HSL was 0.05 µg/mL, which is consistent with the results of biofilm formation (Section 3.6) and spoilage factor production (Section 3.7). Moreover, the expression of genes *SgaS*, *TolC*, *pvdA*, and *LuxR* was significantly increased by 2.15-fold, 1.35-fold, 1.51-fold, and 1.69-fold, respectively, at the maximal supplementation of 0.2 µg/mL (*p* < 0.05). Gene upregulation also explained the stimulatory effect of exogenous C_4_-HSL on biofilm formation, spoilage factor production, and AHLs transduction.

### 3.9. Molecular Docking Analysis

**Figure 10:**
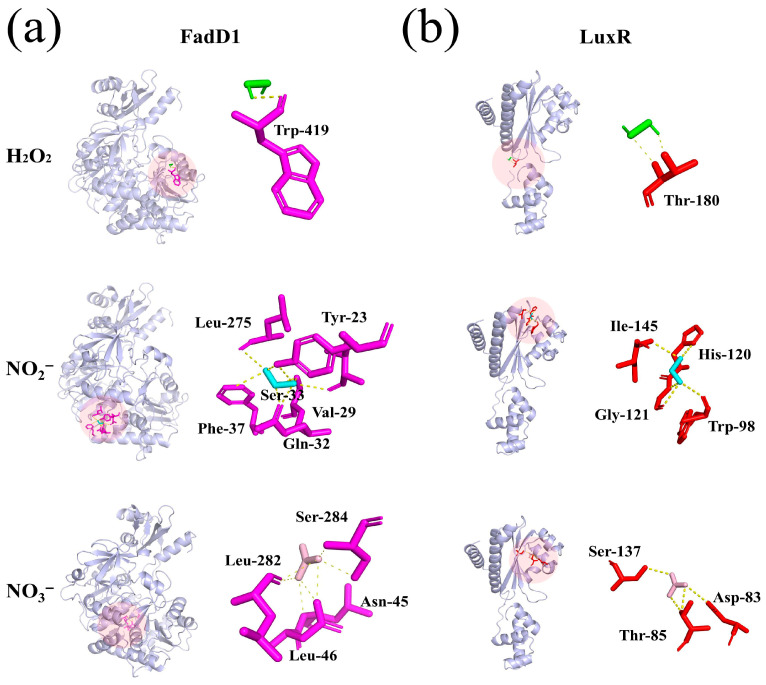
Molecular docking plot of H_2_O_2_, NO_2_^−^, and NO_3_^−^ in PAW with (**a**) FadD1 and (**b**) LuxR.

The binding interactions between the long-lived reactive species of H_2_O_2_, NO_2_^−^, and NO_3_^−^ in the PAW and AHLs synthetic (FadD1) and AHLs receptor (LuxR) proteins were investigated. Figure 10a shows that H_2_O_2_ formed one hydrogen bond with Trp-419; NO_2_^−^ formed six hydrogen bonds with Leu-275, Tyr-23, Ser-33, Val-29, Gln-32, and Phe-37; and NO_3_^−^ formed seven hydrogen bonds with Leu-282, Ser-284, Asn-45, and Leu-46. Figure 10b shows that H_2_O_2_, NO_2_^−^, and NO_3_^−^ formed two, five, and four hydrogen bonds with the amino acid residue of LuxR, respectively. H_2_O_2_ interacted with Thr-180. NO_2_^−^ interacted with Ile-145, His-120, Gly-121, and Trp-98. NO_3_^−^ interacted with Ser-137, Thr-58, and Asp-83. These findings demonstrate that the three long-lived reactive species in PAW can bind to the various amino acid residues of AHLs synthetic proteins and receptor proteins through hydrogen bonding, thereby disrupting AHLs transduction of the QS pathway, which further inhibits biofilm formation and spoilage factor production. Our previous study showed that a PAW-60 treatment downregulated the expression of the key AHLs QS genes, including AHLs synthetic (*FadD1*) and AHLs receptor (*LuxR*) [30]. The molecular docking further presented the interactions of the reactive species and the key proteins at the molecular level. These findings are consistent with the work of Gao et al. [54], who demonstrated that carvacrol binds to the core components (AgrA, AgrB, AgrC, and AgrD) of the Agr QS system in *Listeria monocytogenes* via hydrogen bonding. Moreover, all four gene expression levels of the Agr QS system were downregulated by 20.29 to 99.01%. Ge et al. [10] reported that benzyl isothiocyanate formed pi-pi, pi–alkyl bonds, and van der Waals forces with different amino acid residues of the LuxR-type protein, thus suppressing biofilm formation in *P. fluorescens*. Yang et al. [55] described that the QSI of peptide Ser-Phe formed hydrogen bonds with several different amino acids in the AI-2 synthetic protein of LuxS and the AI-2 receptor protein of LuxP, thereby destroying the AI-2 QS system in *Vibrio parahaemolyticus*.

### 3.10. The Effect of PAW on the Spoilage Potential of PF14 in Fish Muscle Juice

**Figure 11:**
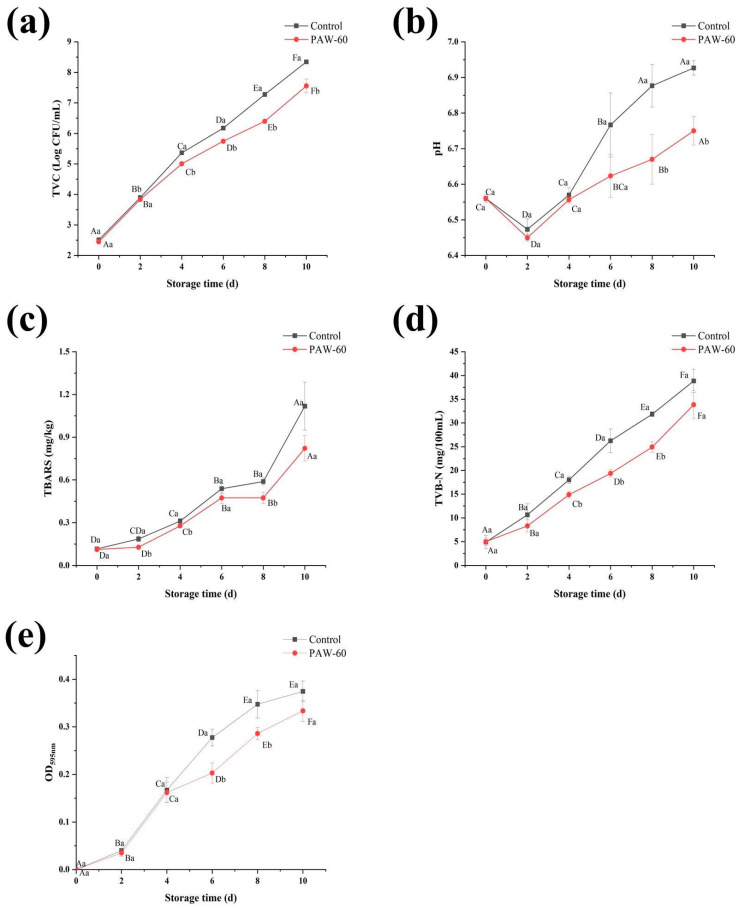
Effect of PAW on (**a**) TVC, (**b**) pH, (**c**) TBARS, (**d**) TVB-N, and (**e**) AHLs production of fish muscle juice during 4 °C storage. Results are presented as mean ± SD (*n* = 3). Different uppercase letters indicate significant differences after same treatment for different storage times (*p* ˂ 0.05), and different lowercase letters indicate significant differences for same storage time after different treatments (*p* ˂ 0.05).

As seen in Figure 11a, the TVC in fish muscle juice increased significantly during the whole storage, and the value of PAW-60 was significantly lower than that of the control (*p* < 0.05). According to the International Committee on Microbiological Practices for Food [17], the maximum acceptable limit for the TVC in aquatic products is 7.00 log CFU/mL. The control and PAW-60 exceeded the acceptable limit on day 8 and day 10, respectively, with a value of 7.23 and 7.55 log CFU/mL. Moreover, the PAW-60 decreased the TVC by 0.79 log CFU/mL compared with the control on day 10. The results indicate that the PAW-60 treatment slowed down the bacterial proliferation during the 10 days of cold storage.

Figure 11b shows that the pH of the fish juice was lower than that of the control after the PAW-60 treatment, and there was a significant difference between the control and PAW-60 on day 8 and day 10 (*p* < 0.05). In addition, the pH values presented a downward trend first (day 2), followed by an upward trend. This phenomenon was attributed to the glycolysis and ATP degradation during the early stage, which produced lactic acid and decreased the pH. Subsequently, the nitrogen-containing compounds in the fish were broken down into alkaline substances with the extension of storage, increasing the pH. The results are consistent with those of Cui et al. [43], who also reported a similar pH trend in tuna fish chunks during 4 °C storage.

The TBARS value represents the degree of fat oxidation, which is also an important indicator for evaluating food quality. Similar to the TVC, the PAW-60 reduced the TBARS, and the values kept increasing during storage, and reached 1.12 and 0.82 mg/kg for the control and PAW-60, respectively, on day 10 (Figure 11c). The TVB-N is an indicator of protein oxidation, and seafood products are considered completely spoiled when it exceeds 30 mg/100 mL [17]. After the PAW-60 treatment, the TVB-N was lower than that of the control during the whole storage, and the control exceeded the threshold on day 8, with a value of 31.85 mg/100 mL, while the value of the PAW-60 was 24.92 mg/100 mL on the same day, and it increased to 33.83 mg/100 mL on day 10 (Figure 11d). The TVB-N results are consistent with the TVC analysis, both of which reached the complete spoilage threshold on day 8 and day 10 for the control and PAW-60 treatment, respectively.

Figure 11e shows that AHLs production kept increasing during storage, and the PAW-60 had a lower value than that of the control, with a significant reduction of 26.85% and 17.14% on day 6 and day 8, respectively (*p* < 0.05). The results also show that AHLs production was positively correlated with the spoilage process of food, and lower AHLs production indicated less spoilage. Lu et al. [56] reported that the QS signaling molecule of AI-2 was involved in the spoilage process of tomatoes.

**Figure 12:**
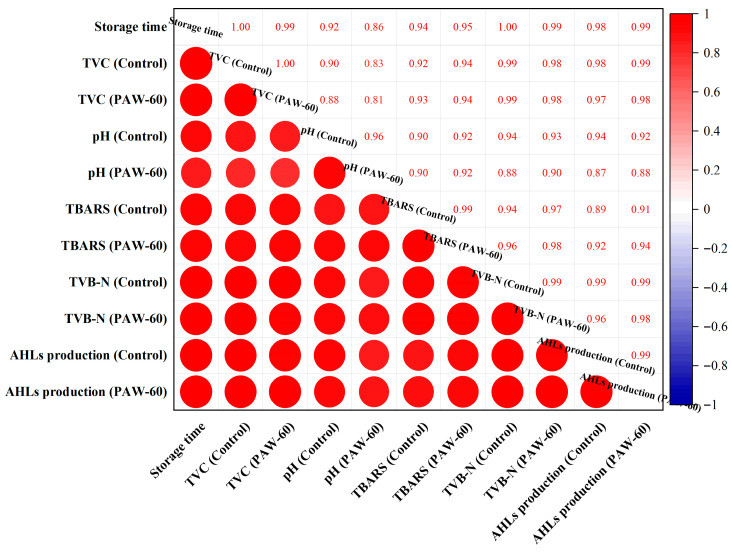
The correlation analysis of the TVC, pH, TBARS, TVB-N, and AHLs production of fish muscle juice and the storage time for both the control and PAW-60.

The results illustrate that the PAW-60 treatment attenuated the spoilage activity of PF14 with in vitro application, resulting in a reduced TVC, fat oxidation, and protein oxidation of fish muscle juice. As shown in Figure 12, the production of QS signaling molecule AHLs was positively correlated with other spoilage parameters, indicating its critical role in the food spoilage process. Though studies on food preservation of under sub-inhibitory conditions remain limited, its bactericidal effects of PAW in preserving various food products have been reported [21,57,58].

## 4. Conclusions

Our study demonstrates that the biofilm formation and spoilage factors in *P. fluorescens* were mitigated by a PAW treatment under sub-inhibitory conditions. There was a negative relationship between the PAW generation time and biofilm formation, protease production, and siderophore production, as well as a positive relationship between biofilm formation and spoilage factor production throughout the 72 h incubation. The selected PAW treatments significantly decreased AHLs production and AHLs activity, except for PAW-30. Meanwhile, oxidative degradation of the dominant signaling molecule C_4_-HSL in *P. fluorescens* was observed, manifested by the formation of a new C=O group following all PAW treatments except PAW-30. This structural alteration aligned with the observed reduction in both AHLs production and activity. Following the PAW-60 treatment, the addition of exogenous C_4_-HSL gradually restored the biofilm biomass, spoilage factors, and gene expression levels of biofilm formation (*SgaS*), spoilage factors (*TolC*, *pvdA*), and AHLs receptor (*LuxR*). The recovery further implied that the inhibitory effects of PAW on biofilm formation and spoilage factors secretion principally resulted from the disruption of AHLs pathway. The molecular docking revealed that the H_2_O_2_, NO_2_^−^, and NO_3_^−^ in the PAW interacted with various amino acid residues of AHLs synthetic protein (FadD1) and AHLs receptor protein (LuxR) through hydrogen bonding. Furthermore, the spoilage activity of *P. fluorescens* inoculated into fish muscle juice was attenuated after the PAW-60 treatment. In conclusion, our study shows that PAW acts as a QSI through three principal mechanisms: (1) suppressing AHLs production, (2) degrading AHLs molecules via oxidative modification, and (3) interfering with AHLs reception. Future studies should involve constructing *LuxI* or/and *LuxR* deletion mutants in *P. fluorescens* to validate the role of QS in food spoilage potential. Additionally, the effects of PAW on the AHLs receptor protein (Lux R) via in vitro expression could help further elucidate the action mechanisms of PAW as a QSI.

## Figures and Tables

**Table 1:** Primer sequences for RT-qPCR.

Gene	Primer	Sequence (5′-3′)
16S rRNA	16S rRNA-F	GGAATCTGCCTGGTAGTGGG
	16S rRNA-R	CAGTTACGGATCGTCGCCTT
*SagS*	*sagS*-F	GCTGAACTCGCTCAGGAACT
	*sagS*-R	TGGCGCCAAACAGAAAATCG
*TolC*	*TolC*-F	AACCGATTTGGTCAGCGTCT
	*TolC*-R	CTTGTTCGTTGACGGCTTCG
*pvdA*	*pvdA*-F	CCTGGTGACCCAGAGTGAAC
	*pvdA*-R	GAGATCACACGCAACGCTTC
*LuxR*	*LuxR*-F	GTGCCAACGCTATGCTGAAC
	*LuxR*-R	TGCGATCCAAACAATGGCAC

## Data Availability

The original contributions presented in this study are included in the article. Further inquiries can be directed to the corresponding authors.

## Abbreviation

Abbreviations and corresponding full terms used in this study.

Abbreviation	Full term
PAW	Plasma-activated water
QS	Quorum sensing
QSI	Quorum sensing inhibitor
C4-HSL	N-butyryl-homoserine lactone
AHLs	Acyl homoserine lactones
AIPs	Autoinducing peptides
AI-2	Autoinducer-2
ACP	Acyl carrier protein
QQ	Quorum quenching
SSO	Specific spoilage organism
TVC	Total viable count
TBARS	Thiobarbituric acid reactive substances
TVB-N	Total volatile basic nitrogen
CV026	Chromobacterium violaceum 026
TSB	Tryptic soy broth
CAS	Chrome azurol sulphonate
HPLC	High-performance liquid chromatography
ONPG	O-nitro-β-d-galactoside
TBA	Thiobarbituric acid
MDA	Malondialdehyde
DMSO	Dimethylsulfoxide
MICs	Minimum inhibitory concentrations
PALA	Plasma-activated lactic acid
